# Global knowledge flows and the psychiatric encounter in Indonesia

**DOI:** 10.1111/maq.12906

**Published:** 2024-12-19

**Authors:** Florin Cristea, Putu Aryani, Yohanes K. Herdiyanto

**Affiliations:** ^1^ Institute of Social and Cultural Anthropology Free University Berlin Berlin Germany; ^2^ Department of Social and Cultural Anthropology University of Münster Münster Germany; ^3^ Department of Public Health and Preventive Medicine, Faculty of Medicine Udayana University Denpasar Bali Indonesia; ^4^ Department of Psychology, Faculty of Medicine Udayana University Denpasar Bali Indonesia

**Keywords:** clinical subjectivity, Indonesia, morality, severe mental illness

## Abstract

In this article, we examine the clinical encounters of people diagnosed with a severe mental illness (SMI). Drawing on more than 1‐year of ethnographic research and interviews in Indonesia, we show that instances of moral self‐reflection occurring in the process of acquiring and appropriating clinical insight emerge at the intersection of heterogeneous discursive regimes. When biomedical notions of health and illness dominate these discourses, they reimagine pre‐existing notions about spirituality and religion. Furthermore, consenting to psychiatric notions of health and illness can create common ground and a sense of shared experience, leading to grassroots movements for the empowerment of the mentally ill, self‐help groups, and other support structures. At the same time, these processes can increase uncertainty and be generative of a culture of blame, as individuals are caught in overlapping and at times contradictory moral systems that each have the potential to strip patients of their moral status.

## INTRODUCTION

Nurul was a young Muslim woman who first experienced symptoms of a severe mental illness (SMI) in high school. These experiences went on for years without her knowing what it was that she was suffering from and without seeing a psychiatrist or going to a mental health clinic. Meanwhile, she visited neurologists and alternative healers who also could not offer any resolution or put a name to her problem. While recounting the onset, she repeatedly referred to her lack of knowledge during those first few years.

The first time was in 2009. But back then I didn't know what it was. I really didn't know. Back then we did not have a diagnosis. We first thought about it in 2009. Then again in 2010. Up until 2016. We thought that I was being possessed. Actually, my mum and my dad thought that. I, myself, I was confused about what exactly this was.

Inviting people to think back to the first time they experienced a severe psychiatric disorder always came with some discomfort. The narratives were mired in contradictions and inconsistencies. Memories were fragmented. We jumped storylines only to return to that initial event a final time. Families intervened and corrected these stories. People's patience wore thin, and remembering an event that fundamentally shattered one's foundation was not a joyful task. Onset, insight, kinship, attitudes toward religion and spirituality, feelings, explanatory models, and other topics anthropologists usually seize during fieldwork, were, at times, incoherent and as diverse as each biography.

What most of those who could recall the initial events had in common, however, was a sense of the world not being quite what it used to be before the onset of the disease, a spurious reality that maintained its structure at the edges but that was threatened to be taken apart by an unknowable force. Our research participants referred to this experience as confusion, feeling unsynchronized, thoughts wandering off, being far away, or simply “not knowing” what was happening. These feelings and thoughts about their health condition accompanied them well into their first visit to a clinic or hospital, sometimes days, weeks, months, and even years after onset.

In this article, we examine the consequences of the clinical encounter (Jenkins & Csordas, 2020) of people diagnosed with a SMI in Indonesia. We trace the tensions that emerge in the interstitial areas between local nonbiomedical representations of disease and global discourses of biomedical psychiatric care, engendered by notions of mental health and illness transported through global mental health (GMH) initiatives and the knowledge flows of global psychiatry. The clinical encounter is a profoundly moral encounter. It is prescriptive of particular kinds of personhood and behaviors (Steinforth, 2017) and proscriptive of what is to be considered appropriate knowledge and health‐seeking (Rose, 2019), altering individual moral perceptions (Lang, 2021) and moral commitments (Biehl, 2013). These moral facets of the clinical encounter do not only emerge in biomedical settings or as part of interactions with biomedically trained practitioners. They are part of the cultural construction of the clinical reality, involving a variety of medical systems, not simply or even primarily the biomedical system (Kleinman et al., 2006[1978]).

In our analysis, we follow the nexus of moral tensions and political contingencies inherent in the interactions of patients, families, and caretakers with particular biomedical and nonbiomedical institutions (Kleinman, 2008). In our case, these were psychiatric hospitals, community health centers, and private organizations that provided psychosocial support for people suffering from an SMI, as well as spiritual complementary and alternative treatment traditions, like *pemangku* [Balinese‐Hindu specialists] in Bali, and *ustaz*, and *kyai* [*Islamic experts*] in Java. We will show that moral clinical subjectivities—instances of moral self‐reflection occurring in the process of acquiring and appropriating clinical insight—emerge at the intersection of heterogeneous knowledge streams that inform a variety of health subjectivities corresponding with diverse healing practices. When these different discursive regimes converge as part of a homogenizing process associated with global psychiatry and GMH, they can aggregate experiences and be generative of blamescapes, following Arjun Appadurai (1996), flows of morally charged discourses, that can challenge the moral status of people diagnosed with a severe psychiatric disorder.

Critical commentaries on GMH initiatives and local applications of global psychiatry have suggested that notions of mental health and illness are being transported from countries of the Global North to the Global South without consideration for local understandings of illness and suffering (Kohrt & Harper, 2008, 11) and where indigenous therapies are of little interest or importance (Sax & Lang, 2021, 9), or considered outright dangerous (Summerfield, 2008, 992). One of the driving forces of this imbalanced relationship between the Global North and the South appears to be a lack of research on SMIs in countries of the Global South (Cohen et al., 2014, 20). While research attention on mental health and illness in the Global South is increasing (e.g., Pinto, 2014, for India; Duncan, 2018, for Mexico; or Tran, 2023, for Vietnam), the movement for GMH remains dominated by knowledge parameters drawn from and established in the Global North (Rose, 2019, 136). This article aims to further address this gap.

Research on the moral experience of people diagnosed with severe psychiatric disorders in Indonesia often focuses on stigma as a phenomenon bound to local notions of prosocial behaviors. Several studies show that stigma commonly follows an inability to achieve harmonious integration with family and community, spirits and ancestors, and is caused by an inability to reach emotional attunement to one's peers by, for instance, not displaying shame [*malu*] when it would be considered an appropriate emotional reaction (Subandi, 2015; Subandi & Good, 2018; Suryani et al., 2011). However, other studies suggest that local moral realities are not necessarily challenged by “immoral” or “deviant behaviors” in the immediate environments of marginalized communities. Transgressions of the destitute ill might already be weaved into the dynamics of the local sociocultural fabric, such as the surrounding networks of friends and families. They can even be engendered by agents that would normally be associated with the person's in‐need support structures, as part of complex emotion‐plays that evoke shame and deference (Stodulka, 2016, 239).

Our contribution to this repository of knowledge is twofold. First, we will suggest that the intersubjective interaction involved in these moral encounters binds local and global notions of what it means to be “good.” Second, we focus on the experiences of people who, like Nurul, transitioned from nonbiomedical to biomedical notions of mental health and illness. This focus on shifting etiology offers a unique perspective on the complex processes involved in acquiring biomedical insight. We will provide this perspective by showing how global psychiatric knowledge flows trickle into local communities and how they shape the illness experiences of people diagnosed with an SMI and their caretakers.

### Research context

This article builds on more than 1‐year of in‐depth research in Indonesia in Bali and Central Java. For a better understanding of the research context, some differences between the two sites warrant attention. First, Bali is dominantly Hindu, whereas Yogyakarta and Central Java are predominantly Muslim. Several research participants from Bali have emphasized that being Balinese means being Hindu, whereas being Javanese was not necessarily associated with being Muslim. Both sites are largely economically dependent on national and international tourism.

Furthermore, in Bali, psychiatrists oversaw community outreach and interventions. In Yogyakarta, psychologists had a more active role in the community. They represented one of the main catalysts for biomedical knowledge outside of clinics and hospitals. Collaborations with religious and spiritual healers, called *pemangku*, in Bali, were relatively common, albeit far from the norm. Whereas in Yogyakarta, professionals regarded such collaborations as being unlikely and considered that the domains of responsibility, the spiritual and the physical, although just as important, should remain distinct.

Structurally, and related to health‐seeking behaviors, the two sites had considerable commonalities. For instance, the psychiatric infrastructure, especially the regional hospitals in Bangli, Bali, and Pakem, Java, developed at the beginning of the 20th century, before independence. This infrastructure was left in shambles by the Dutch colonial government. Despite restructuring efforts—which since independence in 1945 waxed and waned depending on other health or economic priorities (Pols, 2018)—to this day, there are massive regional differences in access to psychiatric care. While it was relatively easy in urban centers like Denpasar (Bali) and Yogyakarta (Java) to access healthcare facilities, it was much more burdensome to do so in rural areas. Both regions had experienced a fair share of natural disasters, which prompted the government to invest in the development of psychiatric care more than in other places in Indonesia. However, this did not always guarantee that patients would seek help from within the available psychiatric infrastructure.

The period between onset and the first visit to a community health center or a hospital could have lasted days, weeks, and sometimes even years. The reasons why our research participants initially avoided psychiatric hospitals and clinics varied. Some lacked transportation, while others did not want to pay for care because they did not have or were not aware of *BPJS*, the national health strategy that aims to provide universal health care in Indonesia. Some others simply did not want to be associated with a psychiatric institution or did not think their ailment to be of a psychiatric nature. Even after obtaining their diagnosis, most patients and families would access biomedical treatments and spiritual and religious healing institutions throughout their therapeutic journeys (see also Lemelson & Tucker, 2017; Marchira et al., 2016). Finally, both sites were places of high ethnic and economic diversity, caused by internal economic displacement, thus making them ideal sites for a glimpse into Indonesia's highly heterogenous social, cultural, and economic environment and into the diversity that is often referenced as one of the major challenges for providing appropriate biomedical care (Agustina et al., 2019).

It is important to note that this heterogeneity was also reflected in the diverse illness narratives we collected. For this reason, in our reporting, we did not deem it necessary to differentiate between Bali and Java. We decided to emphasize cultural differences as they emerged in individual narratives and only refer to them where necessary. The instances remain marginal, as our main focus was on interactions with biomedical knowledge flows which did not vary substantially between the two sites. In other words, many of the differences and similarities we found were engendered by individual experiences with biomedical psychiatry and less by any potential cultural disposition owing to the fact of being Balinese or Javanese. Although we do not wish to claim representativity for the entire archipelago, several of the experiences we describe are similar to accounts from outside of Bali and Java (see for instance Minas & Diatri, 2008, for Sumatra; Broch, 2001, for Sulawesi; or Samuels, 2019, for Aceh).

### Methodology

This article builds on 12 months of in‐depth research in urban and rural Indonesia conducted between 2022 and 2023 and 3 months of exploratory fieldwork from 2019. We obtained access to research participants through previously established professional networks that Putu Aryani and Yohanes Herdiyanto had access to as PhD candidates in Denpasar, Bali, and Yogyakarta in Java, respectively. And by snowballing through preestablished professional networks that Florin Cristea had gained access to during his exploratory fieldwork in 2019.

Our project's main question was how global psychiatric information flows influence local perceptions and understandings of mental illness and how these affect the corresponding emotional and moral milieus of people diagnosed with an SMI. The authors collaborated on data collection separately during different stages of the fieldwork (FC and PA in Bali, FC and YH in Java). We collected 33 recorded interviews with patients diagnosed with SMI, often with the participation of family members. Furthermore, we collected 37 interviews with biomedical practitioners, five with government representatives, seven with representatives of nongovernmental organizations, and five with spiritual practitioners. We used a modified version of the McGill Illness Narratives Interview (Groleau et al., 2006) for the recorded interviews with patients and a separate interview guide for professionals following similar themes. Additionally, we visited several patients and their families, whom we could not interview because their condition was unstable or because they declined to be included in our research.

Given their prevalence and importance in dealing with mental health, a notable limitation of our research sample is the relatively small number of nonbiomedical healers in our study, which does not reflect the variety of local healing practices (see Lemelson & Tucker, 2017, for Bali and Good et al., 2019, for Java). This limitation occurred for two reasons. First, families were reluctant to connect us with the nonbiomedical healers they had worked with in the past, possibly because they associated us with biomedical institutions, which do not always endorse accessing such services. Second, we relied on health professionals to recommend nonbiomedical practitioners whom they knew to have been treating people with severe psychiatric disorders. Therefore, the ones we accessed were to some degree familiar with biomedical nosology and accepted the primacy of biomedical treatments, at least as a first step of intervention after onset. This point is reflected in our findings.

Furthermore, the practices of the recommended alternative healers corresponded to state‐sanctioned religions and, therefore, perhaps enjoyed more acceptance in communities and among professionals than animist healers called *balian* in Bali or *dukun* in Java. For instance, *pemangku* in Bali were not sought just for their healing powers but also because they were considered Balinese‐Hindu experts and usually held functions as temple caretakers. Similarly, *ustaz* and *kyai* held roles as Javanese‐Islamic experts and teachers. Nevertheless, there is a body of literature and, most recently, a documentary by Erminia Colucci and her colleagues (2021) about the relationship of psychiatrists with various types of nonbiomedical healers in Indonesia that has informed the interpretations of our findings.

Finally, the first author regularly visited organizations that provided psycho‐social reintegration programs for people diagnosed with a severe psychiatric disorder. He joined field visits of organizations and professionals who supported patients in rural areas. He attended psycho‐education events, psychiatric workshops, the National Congress of Indonesian Psychiatrists in Medan, North Sumatra, and visited Hindu and Islamic centers that provide care outside biomedical institutions. These micro‐political contact zones were sites where patient subjectivities were constantly negotiated and shaped (Stodulka, 2015).

All participants gave written or verbal consent to be included in this research. The research was ethically cleared by the Ethics board of the Free University Berlin, by the Indonesian National Research and Innovation Agency (BRIN), and by local government offices (Dinas Penanaman Modal). We use pseudonyms throughout the article.

### Global knowledge flows

We situate clinical moral subjectivity in the context of severe psychiatric disorders at the convergence of two distinct but mutually informed streams of knowledge. One flows from the top down, from the global, through the national, to the local. The other flows from the bottom up, from lived experiences of disease and distress, grassroots movements and activists, through NGOs, and into local politics of standardization and representation. Although the two are immutably linked, the first enjoys discursive dominance under the pretext of scientific claims to truth. Institutionalized global discourses infused with the power and authority of evidence‐based regimes of truth and implications for local biomedical institutions and markets have been widely explored by social scientists (Biehl, 2013; Mills, 2014; Rose, 2019). Adhering to this global world of biomedical truths constitutes one of Indonesia's primary modernity projects (Good et al., 2007).

In what follows, we will explore the tensions that emerge at the intersection of global normative discourses and practices, local implementations, and the implications for the moral subjectivities of patients, their caretakers, and the professionals involved in their care.

### The psychiatric encounter

Indonesia has been under international scrutiny for the persistence of *pasung*, the chaining and shackling of people suffering from severe psychiatric disorders (Hunt et al., 2021; Minas & Diatri, 2008), much to the discomfort of professionals, who feel unfairly blamed for the endurance of such practices. Dr. Dewi, a psychologist with extensive experience in international collaborative projects, highlighted that international researchers should be aware that in a context of limited resources, it is difficult to hold a country the size of Indonesia to the same standards as countries in the Global North that are much smaller in population and that have access to more extensive mechanisms of control of the sick population and their caretakers (see also Colucci, 2024).

The problem was not that practitioners were not aware of the challenges of caring for someone suffering from an SMI, but that “[…] the system here is not yet strong. The families often do not have a choice. Hence, they use *pasung*. If they don't, they *[the sufferer]* will run away.” As a case in point, while visiting a family in a village that had confined a male relative, we learned about a small NGO that had replaced the wooden shed with a stone structure that resembled a living room, equipped with a small sofa, a bed, and a radio, as well as access to a small bathroom. Intervening, in this scenario, did not mean releasing him but creating more humane conditions of confinement. Public discourses attributed instances of *pasung* to a lack of education about the nature of psychiatric disorders, and a shared hope was that psychoeducation at a large scale would eventually decrease stigma and render instances of *pasung* obsolete (Arjadi et al., 2018; Budiono et al., 2021).

Indeed, some families and patients regarded psychoeducation positively and considered their recovery a success in part thanks to their ability to acquire more knowledge about their suffering. For instance, Pak Cokot, whose progress several psychiatrists who attended his case described as “extraordinary,” ended up in the psychiatric hospital after an outburst [*ngamuk*] on the construction site where he was working. He became an artist and an activist in the fight against *pasung*, has exhibited paintings in several major cities, and has been invited to psychoeducation events to speak about his experiences. During a highly self‐reflective interview, he explained:

I didn't understand what was happening to me, everything seemed upside down. Unusual. But I didn't know what it was that I was feeling. At that time, I felt, that some people at work used magic [sic] on me. [After calming down] I was allowed to go home. But my thoughts were still far away. Now I know, but back then I didn't know, I was hallucinating. […] Now, we want to educate people. That this is a real disorder, a physical disorder. This is not a supernatural illness. […] Many people in Bali believe that a mental illness is an illness of the spirit [roh]. In Hinduism, we call it Atman. A pure element of God. But when I use mental illness, schizophrenia, the spirit remains right. It is a medical [physical] problem.

Similarly, Eve was first hospitalized in the Bandung Area in West Java, in her early teens, after her friends and family discovered cuts on her body and learned about her history of self‐harm. She also recalled the clarity that followed learning about her diagnosis a couple of years after the initial hospitalization.

[Author Initials]: Has the diagnosis changed the way you look at yourself?

Eve: In a way, yes. But not in a bad way. Because, again, you know … putting a name on this problem. Before that, I didn't know what it was. I didn't know what to do with it. But knowing that, this is something that is not exclusive to myself, this is something that happens in the world. Other people have this too. Other people study about it. And other people find ways to solve this. That gives me the strength to believe that I can do something about it.

Such positive affiliation to biomedical models confirms findings from the psychiatric literature, which suggest that biomedical insight produces better illness outcomes and reduces stigma (Hansson et al., 2016; Torrey, 2019, 47). It diverges to some extent with findings from the critical social sciences and labeling theory concerning how diagnosis is appropriated and embodied, often leading to self‐stigmatizing behaviors and loss of hope (Goffman, 1991; Luhrmann, 2017). Nevertheless, clinicians considered biomedical insight, treatment adherence, and clinical compliance as moral markers signaling one's recovery, irrespective of potential negative implications for their sense of hope and self.

The standards by which progress was evaluated sometimes turned out to be relatively low. We reached many patients thanks to the support from local community clinics. We asked for recommendations about patients who showed good illness progress and had not relapsed over the past 6 months. The patients who were considered to have met these requirements were mostly those who consistently took their medication and caused the least amount of trouble for caregivers and families. This often translated into living in seclusion or not engaging with their environment, and many times barely uttering a sentence. Achieving moral clinical standing, therefore, became almost synonymous with being quiet, which did not always align with the expectations and hopes of patients and their families. This contrast between biomedical and patient expectations is particularly visible in how biomedical professionals and patients envision recovery.

Subandi (2015) suggested that recovery in the Indonesian context, more particularly in Java, comprises three phases: gaining insight, struggling to achieve recovery, and achieving harmonious integration with the community. The biomedical notions of recovery we identified among professionals appeared separated from local notions of social recovery (Subandi & Good, 2018, 36). Flora Cohen and colleagues (2024) have similarly suggested in their scoping review of conceptualizations of recovery in Indonesia, that strictly biomedical ideas—usually transported through GMH and global psychiatric knowledge flows—cannot always be integrated into the Indonesian context, and that spiritual or cultural indications of recovery are often prioritized in communities over clinical notions. Furthermore, as we will elaborate below, psychiatrists and clinicians would frame recovery in their conversations with patients and caretakers by comparing the experiences with SMIs to chronic diseases such as diabetes or high blood pressure. This not only increased uncertainty but also suggested that professionals had little hope for recovery, ignoring a large body of scientific research that argues that recovery is possible despite living with an SMI (Harding, 2024).

**FIGURE 1:**
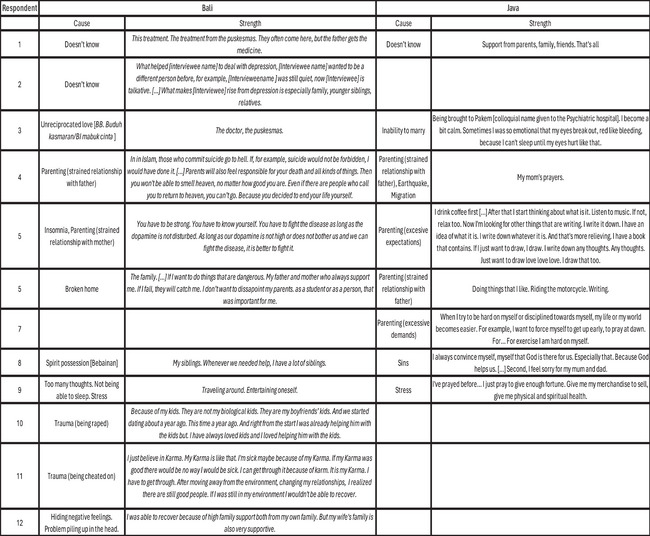
Attributed causes to the disease and recovery.

On the other hand, patients had some notion of having recovered, especially after reaching relatively stable conditions after the acute phases of their disease. In our conversations with them, we tried to determine if there was a relationship between the explanatory models they used and what they considered to have given them strength to recover from their illness. We interviewed 19 patients about the causes of their disease and what helped them overcome times of sickness (Figure 1). Their attending clinicians described these participants as having good insight into their disease, being clinically compliant, adhering to their medication prescriptions, and therefore, having a good illness outcome.

It was therefore not surprising that most answers associated the disease with triggering external events for which respondents considered themselves to have been genetically or neurologically ill‐equipped, corresponding to the biomedical view of disease emergence (*n* = 13). However, reflecting on how they overcame times of sickness, biomedical treatment featured only marginally (*n* = 3). Dominant attributes of local moral economies, such as support via kinship structures (*n* = 6), faith (*n* = 6), and individual ability to endure and overcome suffering (*n* = 4), were considered far more likely resources for strength and recovery. In other words, beyond the psychiatric encounter, moral clinical subjectivities could include characteristics of biomedical insight. However, its potential positive attributes might have remained anchored in patients’ nonbiomedical social worlds.

A divergence did not only occur between global biomedical and local notions of recovery. The language of psychoeducation in clinics and communities and the proposed solutions did not always align with the life‐worlds where these were supposed to be implemented. During outreach events, the solutions included assertive communication with patients and educating oneself. These presentations insisted on the physical nature of psychiatric disorders, dysfunctional brains, and imbalanced neurotransmitters, commonly backed up by slideshows illustrating images of the frontal cortex, colorfully highlighted amygdala, and cited studies and statistics of the WHO. While these presentations may have resonated in formally educated middle classes in university classrooms, we also attended such events in rural central Java, where most participants were women skilled in farming and gardening but with little formal education and of lower economic background.

Clinicians were fully aware of these discrepancies. *Kader Jiwa*, *puskesmas* [community health center] employees or volunteers who carried out community outreach activities at the request of community health centers, were charged with translating this information for the communities they served. Psychiatrists and psychologists would also take it upon themselves to communicate with patients in “culturally” appropriate ways. One recurrent topic of concern was the common practice of blending biomedical and nonbiomedical treatment traditions, which they often attributed to local “beliefs” and “culture.” A few exceptions notwithstanding, professionals were not against complementary spiritual or religious healers such as *pemangku [Balinese‐Hindu specialists] *in Bali, or *kyai *and *ustaz [Islamic experts]* in Java, as long as they did not intervene with biomedical treatments. Denni, a highly active psychologist in rural communities, described coaxing families as a form of negotiation.

We're bargaining with them. But we have to respect those aspects. We don't live where they live. How can we cut it [their relationship]? ‘Ma'am, don't go to a dukun [Javanese spiritual healer]!’ They don't want to accept it. Because that is an alternative that has been around for a long time. […] Of course, we need effective communication to educate them. If we say ‘schizophrenia,’ it is a foreign language, not the mother tongue. The mother tongue is stressed [orang stress], crazy [Javanese: edan], nerves [syaraf], crazy [gila].

The ability to switch between local idioms of distress and biomedical terms was a challenge but also an opportunity to establish oneself as a culturally sensitive practitioner. Especially among practitioners who were engaged in community outreach programs, being able to approach complex medical issues in lay terms separated them from psychologists and psychiatrists who were mainly active from within their private practices or clinics. At the same time, attending to global standards of psychiatric care and drawing from globally established psychiatric units of measurement, reiterated in universities, conferences, workshops, and as part of online seminars, signaled professionals’ connectedness to a globalized medical world governed by evidence and parameters, thoughts and ideas that were not partial to local realities but based on references to scientific truth (Pinto, 2014, 31).

### Psychiatric universalism and Indonesian diversity

Providing evidence for the divergence between global standards of healthcare, usually established in countries of the Global North, and the realities practitioners face at the coalface, usually in countries of the Global South, is a long‐standing tradition in anthropology (Basu & Steinforth, 2017; Read, 2018; Sax & Lang, 2021). One of anthropology's contributions to the study of mental health and illness is situating illness experiences and outcomes within local economic, social, and political contexts (Good, 1997). In terms of local political agendas in Indonesia, few items discursively dominate as much as the emphasis placed on social and cultural diversity. This diversity does not necessarily refer to an immigration‐driven phenomenon (as in *Shattering Culture. American Medicine Responds to Cultural Diversity* edited by Mary‐Jo DelVecchio Good and colleagues, 2011) but to localized diversity: a high social, cultural, and economic heterogeneity, where immigration plays a role but is not its main driving force. This normative discourse provides a counterweight to the homogenizing force of the global psychiatric narrative.

Starting from the country's motto, “Unity in diversity” [*Javanese*: *Bhinneka Tunggal Ika*], diversity is a prevalent issue for political reflection. The archipelago is home to six state‐sanctioned religions, more than 1000 ethnic groups, and more than 700 regional languages, with a majority speaking the national language Bahasa Indonesia. Furthermore, international reports repeatedly spotlight a highly heterogeneous distribution of wealth and inequalities in access to education and healthcare (Chancel et al., 2022).

This cultural and economic diversity also transpired in terms of health‐seeking behaviors and attitudes and understandings of illness. Several instances featured prominently during our fieldwork. First, access to biomedical institutions and medications differed in urban centers compared to rural areas. Some *puskesmas *would prescribe first‐generation antipsychotics, such as Haloperidol, and not, for instance, Risperidone, which has reduced side effects and was usually available in regional hospitals, mainly because *puskesmas* were often restricted to essential drug lists that did not include second‐generation antipsychotics. Whether a patient lived in a village, or the city also determined if a GP, a psychiatrist, a psychologist, or a psychiatric nurse handled the monthly evaluation interview.

Second, explanatory models varied from genes, neurotransmitters, unreciprocated love, and parenting to spiritual punishment from God, use of magic [*santet*], possession, and jealousy. Finally, the socioeconomic situation varied significantly. Most patients we interacted with had no means of income and spent their time at home in isolation. Those who worked usually had low‐paying informal activities, such as directing motorcycles into parking lots or sorting the garbage for recycling. These instances warrant the question of how homogenous biomedical psychiatric notions of health and illness find resonance in such a diverse population. We will address this question next.

### Global knowledge flows and their implications for the psychiatric experience

As previously mentioned, one‐way biomedical psychiatry becomes the dominant narrative for mental distress in any given society is its claim to scientific truth. A particularity in Indonesia was the entanglement of psychiatrists and clinical psychologists to the national project of coagulating a very heterogeneous society. They held this role since the first sparks of the national struggle for independence in the early 20th century (Pols, 2006, 2018). The universal makeup of the human mind implied in biomedical psychiatry could serve this mission through its implication of the “psychological unity of humankind” (Heaton, 2013, 22).

Considering the positive implications of global notions of mental health and illness, why does the psychiatric discourse continuously find itself under the scrutiny of anthropological investigations, much more so than other Global Health initiatives? On the one hand, there is a sense that mental illnesses are cultural to the core, influenced by macro‐social and local conditions of power and meaning, and constituted as distinctive local psychologies (Good, 1997). These might be perceived as being closer to culture than other medical conditions, thus reiterating mind/body dichotomies that have been extensively critiqued in the anthropological literature. On the other hand, there is a genuine concern about biomedical knowledge taking over at the cost of indigenous ways of approaching health and disease, based on uncertain evidence (Davies, 2013; Heinrichs, 2001) and on knowledge mostly collected in resource‐rich countries of the Global North (Bracken et al., 2016). This process has been described as medical globalization (Dilger & Mattes, 2018), biomedical imperialism (Rose, 2019), and even epistemicide (Santos, 2016). A process to which alternative healers in Indonesia were not impervious (Hornbacher, 2021).

Nevertheless, most patients were using alternative and complementary healing. Some specifically dissociated from *balian *and *dukun*, perceived as healers and tricksters, even if they had positive experiences in the past, perhaps because professionals often discouraged such services and described their relationship with *balian *and *dukun *as mutual distrust and disbelief. They sometimes accepted complementary treatments provided by *pemangku* in Bali or *kyai* and *ustaz* in Java. The main difference between these different types of healers was that *balian* and *dukun* were associated with healing traditions dissociated from state‐sanctioned religions, whereas *pemangku, kyai, and ustaz* were considered Balinese‐Hindu respectively Javanese‐Islamic experts. Being perceived as an extension of acceptable religious forms possibly led to increased acceptance among biomedical professionals and translated into forms of collaboration.

Some of the *pemangku* and *kyai* we interviewed trained in the neurochemistry of psychiatric disorders and the importance of complying with pharmaceutical prescriptions. They shared the information they learned with their clients and families. Consequently, attitudes toward spirituality and mental health were re‐evaluated considering disease experience and psychiatric encounters. For instance, prayer was described as a catalyst to focus the mind and to ask for strength from God. Notably, an unfocused mind was considered one of the main attributes of an SMI. Individual attitudes vis‐à‐vis divinity and spiritual forces, while remaining in the domain of religious experiences, would be translated as a source of coping and resilience in the face of mental distress. Muslim patients and their families accepted their struggle as a test from Allah, while Hindu patients would attribute their fate to Karma. In other words, concerning illness experience, we could find little traces of the richness and complexity of Indonesian religious diversity, described to occur even within in‐groups of the same faith (Beatty, 1999; Wikan, 1990).

Moreover, like in the cases of Pak Cokot and Eve, subscribing to the biomedical narrative could decrease uncertainty and improve one's moral autonomy and self‐perception. It created the grounds for shared experiences of mental illness that led to grassroots organizations, support groups, and other self‐help structures like patients and families who regularly attended meetings and events. Such convergence around biomedical taxonomy is not a particularity of the psychiatric encounter. Anthropologists have described similar findings in contexts that did not revolve around mental ill health, such as injuries following nuclear disasters or physical harm (Bradley, 2021; Petryna, 2013). Patients who did not ascribe to the biomedical narrative were less likely to converge around their pain with others and felt, therefore, left alone in their suffering.

However, following the diagnosis, uncertainty could also increase. One contributing factor was the clinician's use of metaphors. Especially concerning medication intake, they compared schizophrenia to diabetes or high blood pressure because of the expected prolonged dependency on medication. Uncertainties concerning medication intake were probably the most common we encountered. When asked whether there was anything they wanted to know about their disease, our research participants had questions about living without medication, if at least they could reduce the doses, or why there was no improvement after taking medication.

Finally, experiences of psychiatric distress were enveloped in a culture of blame (Farmer, 1992). Whether it was professionals blaming families for not providing adequate care, caretakers accusing their sick relatives of not trying hard enough, or patients accusing their families of not caring or not understanding their struggle, cementing moral arguments through blame was relatively common. Probably the most consequential example was mental health professionals accusing alternative and complementary healers of delaying help‐seeking and exacerbating SMI‐associated symptomatology (see also Good et al., 2019). This approach created tensions between biomedical and nonbiomedical healers, between family members who attributed different causes to the disease, and between families and local nonbiomedical healers, whom they would accuse of having taken advantage of them.

Available evidence supports that periods of untreated psychosis can lead to worse outcomes (Howes et al., 2021) and that in Indonesia, visiting nonbiomedical healers prolongs these periods (Marthoenis et al., 2016). However, placing the blame solely on healers disregards correlations between the rapidity of onset and improved care‐seeking and illness outcome, irrespective of whether the received care was biomedical (Good et al., 2019). While our 1‐year fieldwork period would be too short to correlate these findings, we did find that a high number of patients—more than half of those interviewed (*n* = 20) and even more among those that were not stable enough to be included in our research—first experienced symptoms during their teenage years or earlier. We did not find evidence of a more positive illness outcome among patients who visited hospitals and started their psychiatric treatments early.

Another problem with these various blamescapes is that they were generative of multiple moral systems that were not always compatible, and each of these systems could render patients or their families immoral. For instance, relationship and work status represented important moral markers. In the previous section, we mentioned patients that clinicians described as having had a good illness outcome and relatively good insight. Regardless, only a few were in a permanent relationship or had stable jobs, and most worked sporadically in low‐paying day laborer jobs. Patients, therefore, had to provide proof of complying with their medication that could numb them down, but also that they sought ways to contribute to their immediate social environments.

This finding is perhaps unsurprising, considering that previous research in the U.S. suggested that for people who experienced psychosis, stigma tends to persist despite recovery (Jenkins & Carpenter‐Song, 2008). A particularity of our field sites, however, was that at its worst, untethered from social and professional acceptance, the illness outcome could turn into a particular form of “social defeat” (Luhrmann, 2007): refusal to talk. Clinicians were aware of the phenomenon and suggested that being verbally poor [*miskin bicara*] was not uncommon, especially for schizophrenia patients. During house visits, tens of patients barely said a thing. One patient explained that she hoped her silence could hide her disease: *“Unless you speak [ngomong],”* she explained, *“it does not look like schizophrenia.”*

Sri, another participant, was initially diagnosed with schizophrenia and later with bipolar disorder. Not only did she have a good illness progression, but she was also involved with local NGOs to fight for the rights of people diagnosed with a psychiatric illness. In addition, Sri organized support groups for patients and their families in her village and became a *kader jiwa*. She described the mentally ill she met and mentored over the years as people “with a lot of thoughts that get tired of their thoughts quickly. They have many thoughts but are silent.” She recounted her own recovery as an emergence from this silence:

[…] It just changed. Finally, I started speaking with my parents. That is something I went through. It started with silence, then I spoke with friends, then with my family.

## Conclusion

Veena Das locates the depository of knowledge through which people represent illness categories and seek help at the intersection of the concrete experience of illness in the family and local community with the clinical encounter (Das, 2015, 39). Our findings suggest that this juxtaposition only partly captures the complex knowledge landscapes people navigate when confronted with a severe psychiatric disorder. Information seeking and the immediate experiences of disease and illness are part of a web knowledge that transcends the local. While the examples we provide are not exhaustive, we attempted to illustrate, first, how this complex web knowledge corresponding to biomedical notions of mental health and illness established within international structures make it into local communities; and second, how these knowledge flows play out and influence the experiences of patients and their immediate environments.

First, we addressed global knowledge flows associated with global psychiatry and GMH and showed that biomedical professionals and private organizations draw from these flows and align to the (moral) standards of global psychiatry while having to find creative ways to deal with the local constraints of a low‐resource setting. We focused on the psychiatric encounter of patients entangled with these flows and suggested that patients’ moral subjectivities diverge from biomedical notions, evident in how they frame recovery. These differences are, moreover, reflected in the language and the (global) knowledge parameters used and the possibilities of the communities to acquire and implement this kind of knowledge. Furthermore, notions of mental health and illness transported through GMH and global psychiatry have a clear homogenizing force. In Indonesia, this force encounters a political context that values and emphasizes local cultural and religious diversity. Psychiatrists and other clinicians not only participate in this discourse but actively help maintain it. They do so by tolerating and exhibiting respect for—some—local belief systems and associated healing practices as long as they do not interfere with biomedical treatments.

Second, we described the implications of these knowledge flows for the moral clinical experience of people diagnosed with a severe psychiatric disorder. For instance, we showed that apart from the psychiatric encounter, global notions of mental health and illness are transported into local communities by biomedical practitioners in close collaboration with biomedically trained spiritual healers. This collaboration reimagines pre‐existing notions about spirituality and religion to reflect biomedical ideas of mental health. Furthermore, we argued that adopting psychiatric notions of mental health can decrease uncertainty and create common ground and a sense of shared experience, leading to grassroots movements for the empowerment of the mentally ill, self‐help groups, and other support structures. At the same time, the same processes can lead to an increase in uncertainty and be generative of a culture of blame that, at its worst, can push patients into a world of silence.

Future research may determine the extent of the global knowledge flows we describe in this article past and outside the psychiatric encounter. Suparna Choudhury and Slaby (2016, 7) argue that globalized psychiatric notions of health and illness spread beyond specialized frameworks and institutions such as hospitals, law courts, or schools and ultimately reshape and inform everyday practices and values that transform the vocabulary of who we are. Anthropologists have convincingly traced the medicalization of everyday suffering entangled with the spread of psychiatric concepts of mental illness (e.g., depression in Kerala, Lang, 2018; or anxiety disorders in Vietnam, Tran, 2023). Indonesia may provide novel insights given its high economic and cultural diversity and increasing access and adherence to these globalized notions of mental health and illness.
